# The Ethical Significance of Antimicrobial Resistance

**DOI:** 10.1093/phe/phv025

**Published:** 2015-09-30

**Authors:** Jasper Littmann, A. M. Viens

**Affiliations:** Institute of Experimental Medicine, Christian-Albrechts University Kiel; Southampton Law School, University of Southampton

## Abstract

In this paper, we provide a state-of-the-art overview of the ethical challenges that arise in the context of antimicrobial resistance (AMR), which includes an introduction to the contributions to the symposium in this issue. We begin by discussing why AMR is a distinct ethical issue, and should not be viewed purely as a technical or medical problem. In the second section, we expand on some of these arguments and argue that AMR presents us with a broad range of ethical problems that must be addressed as part of a successful policy response to emerging drug resistance. In the third section, we discuss how some of these ethical challenges should be addressed, and we argue that this requires contributions from citizens, ethicists, policy makers, practitioners and industry. We conclude with an overview of steps that should be taken in moving forward and addressing the ethical problems of AMR.

## Introduction

Numerous biological, behavioural, economic, environmental and social factors contribute to the production and propagation of antimicrobial resistance (AMR). After decades in which AMR was almost exclusively a topic for discussion among experts in the medical and microbiological profession, a recent push to broaden our approach and place it more prominently on the political agenda has resulted in wider public awareness of the threat it poses. As a part of this increase in political action and public awareness, the numerous ethical issues associated with AMR are also slowly gaining greater prominence.

AMR has been described as one of the major threats to individual and population health in the 21st century, and national and international organizations have repeatedly underlined the urgent need for action (Davies and Gibbens, 2013; World Economic Forum, 2013; O'Neill, 2014). This year, the World Health Assembly (WHA) passed a new global action plan on AMR and the topic was discussed as one of the priorities for policy action at a recent G7 meeting (World Health Organization, 2015b). The move towards a more substantial response may partly be driven by an improved understanding of the consequences of inaction and its ethical implications. The Centers for Disease Control and Prevention estimate that as much as $20 billion in direct health care costs and $35 billion in lost productivity are associated with AMR to the US economy annually (Centers for Disease Control and Prevention, 2013). A report commissioned by the UK government last year suggests that the death toll of AMR could be as high as 300 million people until 2050, with an estimated total financial loss of up to $100 trillion (O'Neill, 2014). While this calculation includes the effect of resistance not just against antibiotics but also against antiviral drugs, it is certainly indicative of the vast scale and seriousness of the problem.

Given the enormous importance of antimicrobial drugs for the functioning and delivery of modern health care, the progressive exhaustion of effective antibiotics presents health care professionals and policy makers with a distributive dilemma that raises complex moral questions of justice, especially how to fairly allocate antimicrobial resources (Millar, 2012; Littmann, 2014). On the one hand, we may have to restrict the use of antibiotics as far as possible to ensure their continued effectiveness (Littmann *et al.*, 2015). On the other hand, we have not yet managed to ensure the provision of adequate access to antibiotics in many regions of the world, where the price of drugs is often prohibitive for patients and where over-the-counter sales have led to an unregulated and uncontrolled use of antibiotics (Laximinarayan *et al..*, 2013). We are, therefore, faced with a situation in which we have to reduce the excessive use of antibiotics in some regions of the world while ensuring access in others (Heyman *et al..*, 2014). At the same, around half of the world's production of antibiotics are still used in animal and fish farming, which has created reservoirs for resistant bacteria and exacerbates the problem further (Health Protection Agency, 2004; Food and Drug Administration, 2010; Bengtson and Greko, 2014; European Medicines Agency, 2014). Efforts to make progress on these issues require us to raise, confront and enact some difficult ethical decisions that will affect the lives, relationships and personal projects of millions of people.

While much has been written about causes and effects of AMR, the ethical issues of drug resistance have so far—with a few notable exceptions (Aiello *et al..*, 2006; Garau, 2006; Selgelid, 2007; Millar, 2012; Leibovici *et al..*, 2012)—not been considered in detail. More importantly, much of what already exists on the ethical implications of AMR focuses on the way in which drug resistance exacerbates problems traditionally associated with infectious disease control, e.g., the restriction of individual liberty for the sake of public health (Fidler *et al..*, 2007; Battin *et al..*, 2009; Coleman *et al.*, 2010; Enemark, 2013). These are undoubtedly important issues that must be addressed, especially in the context of drug-resistant tuberculosis, where prolonged treatment and decreasing treatment success has led to growing numbers of contagious patients that do not respond to first-line therapy (Singh *et al..*, 2013; World Health Organization, 2014b). However, there are various additional ethical considerations, which a comprehensive response to AMR must address, and which have so far not been adequately addressed by researchers, practitioners and policy makers.

This paper seeks to provide a state-of-the-art overview of some of these current ethical challenges—focusing on three broad questions: (i) why AMR is an ethical issue, (ii) what specific moral questions it raises and (iii) how we should broadly approach the ethical issues raised by AMR. It also functions as an introduction for this journal symposium, which includes contributions exploring various philosophical, political and ethical issues raised by the problem of AMR. This symposium, which is the result of an international, multi-disciplinary workshop on the ethical implications of AMR hosted at the Brocher Foundation in Geneva on March 27–28 2014, seeks to address some of these questions in greater detail and provide a foundation for subsequent discussion. The event brought together experts from different academic disciplines, including medicine, public health, philosophy, economics and law, and provided a platform for discussion and the exchange of ideas across disciplinary boundaries. We are therefore happy to be able to present some of the proceedings of this symposium within *Public Health Ethics* and believe that these contributions offer new and important insights that can help to inform and shape policy-making for AMR. These contributions will be discussed in the context of the wider ethical issues that AMR present.

This paper is divided into four sections. In the first section, we outline some of the reasons why AMR is a distinct ethical issue, and not merely a technical or medical problem. In the second section, we elucidate some of these more general themes and offer a more detailed account of a number of ethical challenges that AMR poses and why they need to be addressed as part of the response to AMR. Many of these issues are also discussed in greater detail in the articles contained in this symposium. Section three discusses how stakeholders should address some of these ethical aspects, and section four proposes steps that should be taken in moving forward.

## Why is Antimicrobial Resistance an Ethical Issue?

AMR is more than a problem that arises as a result of the complications of treating infectious diseases; it is a complex, multifaceted global challenge that affects the environment, human and animal health, agriculture and the economy. Given the multitude of persons, institutions and societies AMR impacts, it presents a distinct and significant ethical issue.

AMR is putting current and future populations at substantial risk of injury, loss and death. It is going to require a redistribution of resources and a balancing of benefits and burdens, which in turn forces us to make a number of individual and collective sacrifices—often for people thousands of kilometres away and for future persons who have not come into existence yet. This will include questions about who is morally responsible for this predicament—and whether ascriptions of blame or sanctions should affect who should bare the costs of this problem. It is also going to require us to intentionally, and sometimes coercively, shape the institutional structures and individual behaviours of governments, corporations, scientists, clinicians and patients, which raises questions about important moral values such as solidarity, liberty, privacy, reciprocity, fairness and the common good.

It is worth exploring some of the broad themes that make AMR a moral issue, and how these considerations contribute to why facing the problem of AMR requires recognition of its ethical implications and their need to be addressed as part of any successful policy response.

### AMR and Risk

The threat of progressing drug resistance puts us at a tangible risk of harm in our life time, and our failure to control and reduce AMR will impose risk on other people and on future generations (World Economic Forum, 2013). The scope of this problem is perhaps easiest to illustrate by the repeated and growing number of warnings by experts in global health and microbiology, who predict the dawn of a post-antibiotic era, should we fail to act quickly and decisively (Davies *et al..*, 2013). While models that predict the impact of AMR on future morbidity and mortality are—by their nature—speculative and dependent on a large number of uncertain variables, there appears to be broad consensus among experts that the effects of AMR are likely to be catastrophic in the near future, if we fail to take appropriate action (Smith and Coast, 2013; O'Neill, 2014). AMR will not only render the treatment of acute bacterial infections more difficult and costly—it will also increase the risk for medical procedures in which antibiotics are used prophylactically, such as surgical interventions or some types of chemotherapy. Allowing AMR to progress unchecked would thus lead to a situation where we might fall short of moral obligations to provide safe medical care, when standard invasive procedures carry high risks of complications or even death. In addition, AMR drastically increases the risk of a return of epidemic and pandemic outbreaks that could be treated with antibiotics in the past (Cars *et al..*, 2008). Already, we are witnessing high levels of morbidity and mortality due to multi drug-resistant and extensively drug-resistant tuberculosis, with drug-resistant typhoid infections becoming more and more common as well (Gandhi *et al..*, 2010; Wong *et al..*, 2015). Current and future generations can therefore expect to be made worse off, should AMR continue to progress. This raises normative concerns about the fair use of resources and whether we have an obligation to bare greater costs or risks to ourselves in terms of food choice, drug consumption and access to particular medical procedures in order to preserve antimicrobial effectiveness for others.

### Responsibility for Acting

It is, however, not only the magnitude of the risk that current and future generations are faced with that creates ethical problems. Due to the speed and scale with which we must react in order to avert a post-antibiotic age, we are also faced with what constitutes a proportional response and, crucially, who bears responsibility to act. While AMR is a complex challenge with numerous causes, it is the broad use of antimicrobials in health care and agriculture that is the driving force behind the emergence of drug resistance (Levy, 2002; Cars and Nathan, 2014). This means that we are unlikely to find a solution to AMR without substantially changing the way we use antibiotics, and reducing the amount we consume. As we shall discuss, drastically reducing the amount of antibiotics we use will have implications for human and animal health, and raise questions about the ethical limits of preserving antimicrobial effectiveness.

However, recognizing the large part that we play in the emergence of AMR also means that we must think seriously about who is accountable for the emergence of drug resistance. If the preservation of effective antimicrobials is in the interest of current and future generations, and indeed their lives depend on it, then we should also hold people blameworthy or sanctionable for the ignorant, unnecessary or wrongful use of antibiotics, or any other practice that is likely to hasten the emergence of AMR. This may mean that we will be morally justified in imposing greater burdens or costs for their contribution to the current state of AMR.

At the same time, however, we must remember that there may be no truly sustainable way of using antibiotics in the long-run, as micro-organisms have shown to be almost infinitely adaptable since the first introduction of antibiotics. This means that our struggle to keep abreast of AMR will most likely be a continuous and vicious cycle of resistance and obsolescence (Aiello *et al.*, 2006; Spellberg *et al..*, 2013). While this does not absolve current generations of their responsibility for their inappropriate use of antibiotics, it should make us reconsider what kind of policy problem AMR represents. If it is not easily solvable, then our moral obligations to future generations may not so much be to fix but rather to manage the problem. We have argued elsewhere that due to its complexity, AMR can be understood as a so-called super-wicked problem, a policy challenge to which no singular solution exists (Littmann, 2014). However, in this symposium, we propose that one way of re-focussing the debate is to consider AMR as a slowly emerging disaster. This would not only account for the scale and severity of the problem for humanity, it would also draw attention to the ways in which a potential post-apocalyptic world would impact on many kinds of human interaction, resulting in uncertainty and increased vulnerability of communities (Viens and Littmann, 2015). Understanding AMR as a slowly emerging disaster highlights a number of ethical and legal challenges that will become increasingly acute. However, we argue that understanding AMR as a slowly emerging disaster also emphasises the need for policies that build resilience, and better prepare us for a world in which fewer effective antimicrobials are available.

### The Cost of AMR

AMR not only uses more resources and increases the cost of health care delivery, by increasing the risk of complications or treatment failure and lengthening the recovery time, it also leads to productivity losses—which are commonly not fully taken into account and have led to an underestimation of the true cost of AMR in the past (Smith and Coast, 2013; O'Neill, 2014). Moreover, there are associated social and economic costs that arise from resultant obligations to care for relatives or friends who have fallen ill from AMR-related disease, which may not be fully incorporated in cost models. Unless drastic measures are taken to prevent or at least slow down a further progression of AMR, health care expenditures associated with drug resistance, both direct and indirect, will continue to grow substantially. This will put pressure on public spending and necessitate a re-allocation of public funds from other areas, sparking concerns about fair resource distribution and health care rationing. However, the development of cost models for AMR creates its own set of ethical problems.

In this symposium, Joanna Coast and Richard D. Smith discuss one of these problems with respect to the use of economic modelling for the prediction of costs associated with AMR. They show that the inclusion of economic models into AMR policy is becoming increasingly widespread, yet the findings of such models are not value-neutral. Depending on their ‘evaluative space’, that is, the parameters they seek to maximize, different models can produce very different recommendations with different distributive effects—a fact that should be noted by policy makers and lead us to discuss more openly which values we hold to be most important in this context (Coast and Smith, 2015).

### Solidarity against AMR

There are no current or future persons who will not be affected by AMR. The risks and costs associated with this problem reflect a shared vulnerability we all have. This fact highlights the need for solidarity between persons, institutions and nation states in responding to AMR. The importance of solidarity is gaining more recognition in the context of bioethics and public health ethics (Prainsack and Buyx, 2011; Dawson and Verweij, 2012; Dawson and Jennings, 2012; Krishnamurthy, 2013; Frenk *et al..*, 2014; Jennings, 2015).

Solidarity is important because it underlines the fact that we will only be able to effectively deal with AMR through collective and collaborative activities, but also because many of these population-level activities will often make it difficult to fulfil some individual-level preferences in order to achieve the public interest that arises out of mitigating AMR. This is not to understand AMR in simplistic terms as involving a conflict between liberty and the common good, or that population-level concerns should always win out over individual preferences. A focus on solidarity seeks to re-orient our ethical focus towards our common interests and vulnerabilities, individually and collectively, and how these considerations should make the distribution of health and risk a joint concern of all levels and sectors of our global society. The solidarity constitutive of a moral community of health justice will confer a mutual recognition, respect and reciprocal concern that requires us to, as Dawson and Jennings put the point, stand up for and stand besides those most affected by AMR (Dawson and Jennings, 2012; Jennings, 2015).

### AMR and Questions of Justice

Following on from the previous point, any solution to the problem of AMR will require a fair balance of benefits and burdens among those affected by it. However, the global burden of infectious disease is distributed highly unevenly and low-income countries are disproportionately affected by AMR. This means that high-income countries will likely have to bear a much larger share of the response, e.g., through developing new drugs and technologies, enhancing surveillance and reporting systems and conducting research in areas that may not be aligned with their current national priorities. At the same time, a response to AMR will also require that we provide better access to high-quality drugs, diagnostic tools and expert care, since AMR is as much a problem of overuse as it is a problem of insufficient access in many low- and middle-income countries (Cars *et al.*, 2008; Laximinarayan *et al.*, 2013; Baker, 2015). AMR therefore does not only raise ethical questions about reducing the use of antibiotics—crucially it will also require a substantial extension of access to antibiotics in many regions of the world (Selgelid, 2007; Laximinarayan *et al.*, 2013). This also involves the quality assurance of drugs that are made available in low-income regions, and the availability of new, patent-protected medicines that may be drugs of last resort. Ethically, this poses challenging questions about how distributive fairness is to be incorporated and balanced with considerations of effectiveness. For instance, if resistance to antibiotics of last resort is likely to emerge quicker the more equally we provide access to them, would this justify restricting access to those who need them most?

## What Ethical Issues does Antimicrobial Resistance Raise?

Having outlined the main reasons why AMR should also be understood as an ethical, and not merely as a technical or medical, problem we will briefly highlight some of the most significant ethical challenges that AMR and our response to it raise.

### The Ethics of Drug and Diagnostic Tool Development

The emergence of drug resistance is an inevitable by-product of widespread antibiotic use (Aiello *et al.*, 2006). To compensate for this, new classes of drugs were regularly developed since the introduction of penicillin. However, after a dearth of new inventions over the past 20 years, the pipeline of new drugs is running dry, and there is now a severe lack of new antibiotics, especially against multi-resistant gram-negative bacteria, which are increasingly becoming difficult to treat (Wenzel, 2004; Morel and Mossialos, 2010; Boucher *et al..*, 2013; Cars and Nathan, 2014). Many campaigns have therefore recently focused on creating incentives to kick-start the development of new drugs and diagnostic tests, such as Drive-AB, New Drugs For Bad Bugs (ND4BB) and the Innovative Medicines Initiative have been initiated over the past years (Goldman, 2012; Rex, 2014; Drive, 2014). In addition, awards like the UK's Longitude Prize are currently dedicated to research on AMR (Rincon, 2014). However, while it is still too early to evaluate the success of these programmes and prizes, it is clear that due to the complexity and the many factors that contribute to AMR, the development of new drugs and tests alone will not be enough to substantially slow down or even reverse the effects of drug resistance. The dilemma we face is thus that the more extensively we use antibiotics, the faster we will create antibiotic resistance.

From an ethical perspective, the lack of new antibiotics poses a number of problems. Perhaps most pressing is the question of who bears responsibility for the development of new drugs and tests, and how these should be made available, once they enter the market. Since both the burden of infectious disease and the prevalence of AMR are disproportionately higher in low- and middle-income countries, there is an urgent need for the provision of low-cost, high-quality drugs and tests to be made widely available. Yet, these countries are also least likely to be able to finance the development of new diagnostic and therapeutic agents, or pay the high prices of novel, patent-protected drugs. There is thus an obvious conflict between medical needs and the current pharmaceutical business model, which seeks to recoup investments into upfront research and development costs through high price and high-volume sales throughout the period of patent protection (Outterson *et al..*, 2011). Since many antibiotics are only prescribed for acute infections and new drugs will be reserved for instances where older antibiotics are no longer effective as a result of AMR, pharmaceutical corporations have been reticent to invest into their development (Horowitz and Moehring, 2004; Aiello *et al.*, 2006). A number of alternative models of funding have recently been suggested, which include, among others, the funding through the Health Impact Fund model and a delinkage of research incentives (Kesselheim and Outterson, 2010; Outterson *et al.*, 2011, 2015; So *et al..*, 2012). These models seek to develop alternatives to the traditional pharmaceutical funding model, where profits are realized through patent-protection. However, none of these models have so far successfully been implemented.

Given the enormous burden of AMR, the lack of implementation raises questions about obligations that high-income countries have towards the rest of the world when it comes to developing, and providing access to new antimicrobials. This must inevitably include a discussion of the reasonableness of insisting on patent protection and high prices for new and essential drugs. Moreover, the uneven global distribution of some infectious diseases means that diseases more commonly found in low-income countries, such as tuberculosis, receive too little funding relative to the mortality and morbidity they cause. An ethical discussion of drug and diagnostic tool development must therefore also consider which research areas should be prioritized.

Finally, the production process of antibiotics itself raises a number of ethical problems, including the substantial environmental impact and pollution caused by dumping by-products into wastewater, thereby affecting rivers and groundwater supplies and ultimately contributing to the emergence of AMR (Brown *et al..*, 2006; Larsson, 2014). An ethical policy response to AMR must focus on all aspects of the drug development process, not just the end products.

### The Ethics of Antimicrobial Stewardship

Since over- and misuse of antibiotics are one of the key drivers for the emergence of AMR (Cars and Nathan, 2014), there has recently been a push towards greater antimicrobial stewardship, which improves prescribing and reduces the wasteful use of antibiotics (e.g., as treatment against viral infections) and to reduce antibiotic consumption as much as possible (Gerber *et al..*, 2013; Centers for Disease Control and Prevention, 2014). For animal use, guidelines and laws that ban the use of antibiotics in low dosages as growth promoters have been implemented in many countries (Casewell *et al..*, 2003; Food and Drug Administration, 2010).

Considerations, such as risk, cost and solidarity, provide all of us—individuals, institutions and corporations—with normative reasons to be good stewards of the effectiveness of antimicrobials. It is not necessarily clear, however, what it means to be a good steward of antimicrobials. There has been a greater focus on the role of stewardship in relation to other public health problems (Saltman and Ferroussier-Davis, 2000; World Health Organization, 2000; Jochelson, 2005; Nuffield Council on Bioethics, 2007; Brownsword, 2009), as well as some critiques about particular ways of framing stewardship (Dawson and Verweij, 2008; Coggon, 2011), which give us reasons to be careful how we should understand the concept of stewardship applied to AMR.

Much of the guidelines on antibiotic stewardship focus on technical considerations—such as improving infection prevention and control, optimising prescribing and consumption practices and developing new diagnostic and therapeutic interventions (Dellit *et al..*, 2007; Centers for Disease Control and Prevention, 2014)—but neglect to consider the moral questions underpinning and guiding what a good steward of antimicrobials should do. After all, being a good steward of antimicrobials is not just about one’s ability to maintain the effectiveness of antimicrobials but knowing *why* one should go about doing so. For this, we must recognise that acting as an antimicrobial steward will often involve making important value judgments. Should the good steward prioritise health above all other possible goods? Should the good steward provide everyone equal access to antimicrobials or can preference be given to some over others? Should the good steward prioritise individual rights and autonomy or collective interests? These questions will be particularly pressing in settings where stewardship does not only entail the elimination of entirely wasteful antibiotic use—for instance, when treating viral infections—but also the restriction of access to beneficial treatments. It has been argued that, given the dire consequences of AMR, it can be ethically justifiable to restrict antibiotic use to instances where their use prevents a substantial risk of irretrievable harm (Millar, 2012). This would mean that a physician may have to expose patients to higher risks of complications, a longer duration of illness, or an increased risk of mortality if the expected benefit of immediate antibiotic therapy is not viewed as being substantial enough. This dilemma between the need for a responsible and restrictive use of antibiotics on the one hand, and physicians' obligations to their patients on the other is an ethical challenge that requires urgent attention from policy makers. A call for effective and expansive antimicrobial stewardship will inevitably imply a restriction beyond the mere avoidance of waste. However, as long as physicians are not provided with clear guidelines on when antibiotic use can be rightfully withheld, they face a severe ethical dilemma in their everyday practice (Kollef and Micek, 2014). Simultaneously, the creation of guidelines alone is unlikely to solve all ethical concerns related to the rational use of antibiotics.

Being stewards of antimicrobial effectiveness also raises complex questions about what kind of goods antibiotics are and how we should regulate their use. The emergence of AMR has often been linked to different types of analogies, such the ones used by microbiologist John Conly who compared the challenge to 'overfishing scenario[s], to cattle overgrazing the grass of the commons or to deforestation on Easter Island, which led to the population dying out’ (World Health Organization, 2010). How we frame the discussion of AMR will impact on which allocation and regulation models should be established. For instance, if effective antibiotics are seen as a public good, their overuse may be likened to the tragedy of the commons scenario (Hardin, 1968; Laxminarayan and Malani, 2007; World Health Organization, 2010). This would suggest that taxation models, which account for externalities of antibiotic use could be useful and justifiable. An alternative suggestion is to view the use of antibiotics as akin to carbon emission. In such a scenario, there might be scope for licenses for antibiotic use that mirror carbon trading schemes (Anomaly, 2010). Elsewhere, we have argued that some of these analogies fail to capture the complexity of AMR and that analogical reasoning, that is the transfer of solutions from one policy field to another, may therefore sometimes be inappropriate (Littmann, 2014). However, irrespective of which model is ultimately adopted, the way in which we frame AMR as a policy problem will have serious consequences for the type of stewardship policies that will be recommended. From an ethical perspective, this is of great importance, because different models of resource allocation, property rights and risk sharing are likely to affect different social groups, thereby raising questions about distributive fairness (Millar, 2011).

Finally, we must recognise, that there cannot be a singular concept of what good antimicrobial stewardship entails. Given the huge discrepancies between access within health care systems, burdens of disease and available resources, what constitutes good stewardship will be highly context-dependent. While this will require the development of different approaches to antimicrobial stewardship, each resultant stewardship frameworks must address citizens, health care workers, government and industry in order to be ethical and effective.

### The Ethics of Ignorance and Behaviour Change

The present state of awareness of the general public about the causes and severity of AMR remains quite poor—even in regions where public awareness and education campaigns have been held (European Commission, 2010; World Health Organization, 2015a). For instance, a significant proportion of the general public do not know that antibiotics are ineffective against viruses and that a full course of antibiotics should be taken and not shared with others.^1^

The general public also continue to practice poor infection control practices, such as adequate hand washing (Aiello and Larson, 2002). We are getting to the point where this ignorance, and frankly in many cases laziness, is inexcusable because it makes it more likely that AMR will continue to increase.^2^

It is possible to increase understanding and motivate behaviour amongst individuals and groups towards ways of acting that reduce their contribution to AMR through various behavioural techniques. There is little discussion, however, about how far we should be able to go in motivating people through these various interventions. Given the general ineffectiveness of AMR public awareness campaigns thus far, we need to think about whether more interventionist, and possibly even coercive, approaches to reducing antimicrobial misuse and poor infection control practices would be permissible. Considering what is at stake, we need to examine whether the use of more behaviour-changing interventions, which seek to guide choice through providing better incentives or disincentives or actually restricting choice in some cases, would be ethically acceptable.

A robust AMR response policy will likely need to include various education and behaviour-changing interventions that seek to improve the level of knowledge and inaction surrounding AMR. These interventions should remind citizens of the obligations they possess—as well as assisting them in meeting their obligations. Citizens have an obligation to educate themselves about antimicrobials—from their use in personal and household products, to consumption of agricultural products treated with antibiotics to how they use antibiotics. Citizens also have an obligation not to infect others, making sure they stay home and adopt appropriate protective practices when interacting with others when ill (Harris and Holm, 1995; Verweij, 2005). Finally, citizens may also need to become more involved in lobbying elected officials and industry to undertake the changes necessary for everyone to actively contribute to reducing the effects of AMR.

One further area where a lack of awareness and individual behaviour are becoming factors for the spread of AMR is the effect of travel and health tourism on the spread of drug-resistant bacteria. In this symposium, Michael Millar discusses the connection between health tourism and AMR and outlines its ethical challenges (Millar, 2015). Health tourists are at greater risk of contracting drug-resistant bacterial infections than other travellers, and in some instances treatment or decontamination may not be possible. This may not only threaten the health of the patient, but also contribute to the international spread of AMR. One response to this has been to suggest the establishment of travel advisories that may issue recommendations to avoid certain regions for elective surgery. Such recommendations have been rejected by some, as they may carry grave consequences for international trade and local economies. However, Millar argues, these are insufficient grounds for failing to inform patients of potential risks and he urges that greater transparency about risks from AMR should be provided by health authorities.

### The Ethics of Agricultural and Farming Practices

In light of the fact that up to half of the worldwide annual production of antibiotics is designated for non-human use, the current debate on the ethics of AMR is heavily and disproportionately focused on the use of antibiotics in humans. While this focus reflects the traditional discourse in medical ethics (Battin *et al..*, 2009), it must be acknowledged that there are a broad range of ethical issues related to the use of antibiotics within agriculture and farming. Most obviously, this relates to issues surrounding animal welfare, specifically because factory farming in its current form is only possible due to the widespread use of antibiotics, not just as an acute treatment option, but crucially also as a prophylaxis for transmission (Rollin, 2001; Anomaly, 2009). Over the past decades, many countries have established bans on the use of antibiotics as a growth promoter in animal farming (Food and Drug Administration, 2010). However, the total volume of sales of antibiotics for agricultural use remains high, and we have yet to fully understand which effect this use has on the emergence of AMR in the environment (European Medicines Agency, 2014). Greater consumer awareness for the use of antibiotics in farming, as well as stricter guidelines on the use of drugs may help to reduce the overall consumption of antibiotics in the agricultural sector. However, they are also likely to have substantial effects on farmers, many of whom will be unable to keep their production output at the same level, if antibiotics are less widely available.

Ultimately, this also raises concerns of fairness over the availability of affordable meat products and produce for lower income groups in society. While neither of these aspects appear to weigh heavily enough to justify the continuation of current practices, we should nevertheless recognise, who will be disadvantaged by proposed policy changes, and discuss what kind of subsidy or compensation may be warranted.

In this symposium, Jonny Anomaly considers some of the ethical implications of factory farming, which continues to be one of the major sites of antibiotic use. He argues that while factory farming has made animal products much more affordable for the consumer, it also creates at least three moral problems, namely, the spread of pathogenic viruses, the diffusion of antibiotic-resistant bacteria into the environment, and the suffering endured by animals in modern farming facilities (Anomaly, 2015). Anomaly argues that the risks associated with the emergence of new viral diseases and the creation of reservoirs of resistant bacteria, as well as the undeniable cruelty towards animals in factory farming, should lead us to revise current industry standards. This includes not only the improvement of living conditions for farm animals, but also the elimination of animal growth promotion through sub-therapeutic dosing of antibiotics. Such changes may come at a cost to both the producer and the consumer but, Anomaly argues, these costs are not a sufficient reason for failing to act.

### The Ethics of Priority Setting and Resource Allocation

Given the complexity and multi-causality of AMR, there are various policy options we can—and must—reasonably pursue, in order to slow the progression of drug resistance that raise ethical questions about fair priority setting and resource allocation processes.

In making AMR a social, political and medical priority, this will likely mean diverting resources from other health and non-health concerns in order to provide more support to AMR mitigation activities. When making AMR a priority, we need to keep in mind that since different interventions will have very different target audiences, a focus on, for instance, the development of new drugs raises the questions which pathogens we should target and who stands to benefit from this, where those benefits will be accrued and what opportunity costs there are. While a focus on drugs against multi- and extensively resistant strains of tuberculosis would help to address one of the major health burdens in many low- and middle-income countries, the development of new antibiotics against highly resistant strains of nosocomial infections is likely to be of greater interest in places with developed and advanced health care systems. Appropriate solutions will thus often be highly context-dependent—and may not always be transferable to other regions or countries. Often, this is likely to be a matter of pricing—a complex new diagnostic tool for AMR or the development of new therapeutic options may be too expensive or simply unfeasible for many low-income settings. In addition to concerns about the global distribution of research outcomes, we will have to think about the relative weight we give to the development of technological, social and ecological problems in the context of AMR—and the ethical issues this raises.

Another perennial issue within priority setting and resource allocation discussions concerns the rationing of antimicrobials, both in terms of reducing overall consumption of antimicrobials where possible but also deciding when to limit new antimicrobials as drugs of last resort. If part of our strategy to preserve antimicrobial effectiveness will involve targeting the provision of antimicrobial drugs, what allocation criteria should be used? This raises ethical questions of whether such rationing decisions should be made on the basis of a cost–benefit analysis focusing on considerations of effectiveness or whether our allocation criteria should also including considerations such as need, social value and equality of access. Beyond these substantive considerations, there also exist procedural questions, such as whether allocation decisions should be informed or guided by public engagement and deliberation in order to be considered ethically acceptable. Further questions will also need to be addressed with respect to how we want to go about rationing antimicrobials, for instance, whether it is best achieved through clinical practice guidelines and how ethical resource allocation criteria should be incorporated within these guidelines.

### Obligations to Future Generations

In addition to expanding our scope of care and concern beyond our own citizens and national borders to low- and middle-income countries, we also need to recognise that our current actions and policies will affect our microbial environment to such an extent that we have to include both current *and* future populations as part of our AMR response. The possibility of an impending post-antibiotic era means that future generations face a risk of being significantly worse off if bacterial infections can no longer be treated effectively, and it raises questions about the obligations we have to future people to preserve effective antibiotics (Leibovici *et al.*, 2012). While there is no straightforward answer to these questions of intergenerational justice, they are certainly complicated by the fact that to preserve antibiotic effectiveness for the future might mean that we will have to significantly reduce our own use of antibiotics. It has been suggested that it is defensible to place patients at some additional level of risk in order to preserve effective antibiotics, and to withhold treatment in cases where antibiotics merely offer a small benefit (Millar, 2012). Yet, such scenarios imply that a given risk for the present patient would be accepted for an uncertain positive effect in the future. It remains an important ethical question within our AMR policy response whether persons who do not currently exist can make claims on current persons, and on what basis such claims would be justified. Whether or not we find such a trade-off acceptable—and under which conditions—will largely depend on what conception of rights of future people we endorse, and different normative theories will come to vastly different conclusions about the duties we owe to future human beings (Macklin, 1981; Partridge, 1990; Mulgan, 2006; Gosseries and Meyer, 2009).

### Health Infrastructure, Social Determinants of Health and the Environment

One person, intervention or sector will not mitigate the problem of AMR. It is going to require not only intersectoral activity between clinical medicine and public health, but wider involvement outside of traditional health-focused institutions and organisations. It is also going to require us to examine one of the fundamental causes and drivers of AMR: our inability or unwillingness to provide the basics to the world’s poor (Okeke, 2010). This will mean linking policy work to the existing rich academic debate on what we owe the global poor and which ethical principles should be guiding national and international action in this area (Rawls, 1999; Risse, 2005; Pogge, 2007).

While it is, of course, important to invest in practices, technologies and drugs that will directly seek to interfere with microbe’s resistance mechanisms, we also need to focus on the basics that contribute to all people living healthy, capable and flouring lives for its ability to reduce AMR (Powers and Faden, 2008; Venkatapuram, 2013; Gostin, 2014). We need to continue investing in the infrastructure and personnel that are required to ensure that all individuals have safe and reliable access to functioning health care and public health systems. This not only will have a direct affect on AMR mitigation (Maralles, 2010), but will also be beneficial to other related global health problems, such as malaria, tuberculosis and Ebola. Additionally, we must continue to place greater emphasis on upstream interventions focusing on the social determinants of health, as well as the environmental factors that contribute and sustain resistance, including the conditions that allow for new and re-emerging infectious diseases.

## How Should We Approach the Ethical Issues Raised by Antimicrobial Resistance?

With greater clarity as to why AMR is itself a moral problem and the number of significant ethical issues it raises, we are still left with the question of how we should respond. In particular, we will want to ensure that our response has normative legitimacy, as well as subject this response to critical scrutiny as it proceeds. In both theoretical and practical contexts, we need to be able to provide and defend the normative reasons that justify the use of political, public health and medical power that will seek to shape our choices and behaviours, redistribute resources and impact our opportunities and capabilities in responding to AMR. This will involve many people and institutions contributing to the development of solutions, policies and critiques in this area. While we do not have the space to defend substantive ethical solutions, we propose that, in moving forward and responding ethically to the problem of AMR, input and effort is needed from at least four areas: (i) ethics, (ii) policy, law and regulation, (iii) public health practitioners and health care workers, and (iv) civil society and industry.

### Ethics

Ethicists have so far largely ignored the issue of AMR. This is problematic for a number of reasons. First, in light of the projected consequences, AMR is one of the major threats to human health in the 21st century. Ethicists, especially those working on bioethics and public health ethics, should arguably be addressing the relevant issues that will affect millions or potentially billions of current and future people across the globe. A greater involvement of ethicists will also be necessary, because—as we have outlined in this paper—AMR creates numerous ethical challenges that are interesting in their own right. Some of them have been touched on in previous contexts. Here, ethicists can help with the transfer of knowledge and expertise.

There are also ethical issues that arise in the context of AMR, however, which require new ideas and analysis. Much of the research that is needed in this area is complicated by the subject matter and the seemingly infinite contributing factors. AMR is thus not a problem that lends itself to the straightforward application of well-established basic principles of bioethics. As a result, further work will be needed from moral and political philosophers on translating agent-centred moral concepts to the population-level and elucidating the relational and socially embedded normative concepts that will underpin the AMR response, such as solidarity, reciprocity, stewardship, collective harm, trust, community, health justice and the common good. But in spite, perhaps even because, of this input from ethicists will be necessary to not only map out the challenges at hand, but also to suggest, how they can best be applied in practice. In addition to conceptual work that aims to clarify and analyse, the fruits of this moral and political thought must also be addressed in policy terms, as well as in public health and medical practice.

### Policy, Law and Regulation

Much of the response to AMR will be approached via the use of governance mechanisms within both public and private institutions, especially instruments such as policy, law and regulation (Fidler, 1998; Sage and Hyman, 2010; Fox, 2011; Anomaly, 2013). Going forward, it is going to be exceedingly important that these mechanisms and instruments recognise and address the many ethical issues involved in responding to AMR. These issues also have to be addressed in a way that has normative legitimacy—not only do we want our governance mechanisms to be morally justified, we also know that being viewed as morally justified increases the likelihood that people will comply with law and policy (Tyler, 2006).

The progression of AMR in many countries suggests that current policy efforts are falling short of the goal to halt or even reverse the effects of AMR (World Health Organization, 2014a). The WHA's recent call for the development of national response plans to AMR is a crucial first step in extending and improving existing legislation. However, it will be important that new policies take into account the ethical challenges posed by AMR. There is also an ongoing discussion about the use of international legal instruments to create agreements that could help reduce AMR (Anomaly, 2010; Hoffman *et al..*, 2015). These proposed legal frameworks could offer another promising avenue for placing the importance of ethical considerations in the context of AMR more firmly on the agenda. While some of the ethical challenges discussed in this paper may be too specific to be the focus of macro-level policy planning, there are a number of areas, where regulators and policy makers could help in reducing normative tensions in our response to AMR.

First, as we discussed earlier, there is a clear need to acknowledge and address the fact that existing policy proposals imply a prioritisation of some interventions over others. How such a prioritisation is arrived at should be subject to open, transparent and accountable discussion. Secondly, policy and lawmakers have a responsibility to help ensure the reduction of antimicrobial misuse. These efforts will also need to be sensitive to the social, political and economic contexts in which AMR efforts are directed. The ethical analysis must be sensitive to, for instance, resource availability and drivers of inappropriate drug use in different regions of the world. The extent to which this is possible will be highly context-dependent; for instance, in countries where antibiotics are available without prescription more fundamental changes will be necessary. However, recent research suggests that lack of appropriate governance is one of the key driving factors in driving the emergence of AMR (Collignon *et al..*, 2015). This would suggest that greater involvement from policy makers and more stringent regulation and oversight in the area of AMR could significantly reduce the burden of drug resistance. Finally, health policy makers play a crucial role in determining how AMR is framed in policy terms. Rather than describing it as a purely medical challenge, policy makers should seek to promote greater collaboration between sectors and research areas to better address AMR as the social challenge it is. Similarly, by focussing on the aspects of mitigation and management, rather than creating an unrealistic and false hope of an imminent technological fix, health policy makers and regulators can help in managing expectations.

### Health Care Workers and Public Health Practitioners

As prescribers or dispensers, medical practitioners, health care workers and pharmacists play a crucial role not only in regulating access to antibiotics, but also in educating patients about AMR. This does not only pertain to a general understanding of drug resistance, but must also involve awareness of own prescribing or dispensing behaviour on the emergence of AMR. However, this requires that they themselves are sufficiently trained and aware of the challenge. Public health practitioners will also play an important role in this effort. Their focus on AMR at the population-level and ability to use the powers and resources of the State in these efforts will often involve navigating between societal and individual interests. If healthcare workers and public health practitioners are unaware or feel unable to address the ethical dilemmas that will arise in responding to AMR, they cannot be expected to respond appropriately. Ethics education, training and guidance need to be provided to these professionals to help them navigate the moral issues they will encounter.

### Civil Society and Industry

AMR strategies predominantly originate in governmental bodies and, as such, tend to focus on patients, professionals and public institutions. Nevertheless, we also cannot forget the role of civil society and industry—particularly in those areas that are not, at least currently, under some regulatory framework. We are going to require more public engagement and citizen action to ensure the success of our response. Not only can this involvement help in identifying the support and limits citizens will be prepared to accept in order to assist in AMR mitigation activities, but it also increases the likelihood of compliance with such activities when they are developed with input and buy-in from the wider community. Industry also needs to be more collaborative and willing to assist to a greater extent without waiting for government regulation to push them further. Current pricing strategies for antimicrobials, especially for drugs of last resort and new medicines that make them unaffordable for a majority of the world's population, would be a good place to start. Showing more concern and solidarity in these matters can only help industry’s public relations and stave off the feeling that increased regulation of industry should be undertaken to force them to do more.

## Moving Forward

Successful responses to the problem of AMR will not only be a scientific or medical undertaking, it must also be an ethical undertaking. This means that an effective AMR mitigation strategy needs to be informed and guided by ethical analysis. As we have shown in this paper, every level of an AMR response strategy will inevitably involve making decisions with ethical implications. Reducing the use of antimicrobial drugs in humans and animals means instituting behaviour-changing interventions and restricting their choices, which are likely to limit preferences and potentially subjecting people to elevated risks of complications or infection and financial costs. Improving surveillance and reporting systems increases concerns about confidentiality and privacy. Preventing and controlling the spread of drug-resistant infections, especially if we have AMR-related epidemics/pandemics, can involve the increased use of restrictive measures, raising questions about constraints on liberty and human right derogations. Promoting research and innovation into different preventative, diagnostic and therapeutic interventions will require us to make funding and allocation decisions that prioritise AMR over other important projects and policies. What these examples entail for policy makers, practitioners and researchers alike is that ethical decisions in AMR policy cannot be avoided—and if such policies are to have any kind of normative legitimacy, they can no longer be ignored either.

While the development of more extensive national and international responses to AMR is encouraging, it will also be hugely important to ensure that the underlying ethical issues will be addressed in the process. The fact that many countries will now begin to develop national response plans to AMR after the WHA, or make updates to existing ones, represents a great opportunity for the ethical significance of AMR to be included more widely into policy making, practice guidelines and training programmes. We believe that the articles in this symposium make a strong case for including ethics, which has not been traditionally associated with being central to infectious disease and global health policy, into the development of the policy responses to AMR that are so urgently needed.

## Notes

1. What complicates the implementation of antimicrobial stewardship further is the fact that while many physicians regularly prescribe antibiotics, the worst effects of AMR are often only visible to specialists or hospital staff. There is thus a disconnect between cause and effect, which may explain why physicians are often aware of the problem of AMR but focus more on the immediate benefit of the treatment to their patient, than on the effect it may have on the community and the emergence of AMR, or may indeed believe that they themselves do not contribute to the problem at all (Metley *et al.**.*, 2002; Simpson *et al.**.*, 2007; McCullough *et al.**.*, 2015). At the same time, as we outlined earlier, many patients continue to be unaware of the causes and effects of AMR. This reduces the adherence to antibiotic treatment regimes and increases the chance that patients will self-medicate or interrupt a course of treatment. This problem is obviously worst in countries where antibiotics are available over the counter and are administered without previous examination by a medical professional (Heyman *et al.**.*, 2014).

2. Though, recent work is beginning to show success with children (McNulty *et al.**.*, 2011; Lecky and McNulty, 2013).
